# The complete chloroplast genome sequence of the endangered species: *Ephedra rhytidosperma* (Ephedraceae): insights into genome features and evolutionary relationships

**DOI:** 10.1080/23802359.2025.2582546

**Published:** 2025-11-05

**Authors:** Xiulian Liao, Jinfeng Niu, Yan Wang, Yuqing Wei, Lei Zhang

**Affiliations:** a School of Biological Science & Engineering, Key Laboratory of Ecological Protection of Agro-Pastoral Ecotones in the Yellow River Basin, National Ethnic Affairs Commission of the People’s Republic of China, North Minzu University, Yinchuan, China; b Ningxia State-owned Forest Farms and Forest Tree Seedling Work General Stations, Yinchuan, China

**Keywords:** *Ephedra rhytidosperma*, Ephedraceae, chloroplast genome, phylogenetic analysis

## Abstract

*Ephedra rhytidosperma* belongs to the genus Ephedra (Ephedraceae) and plays a significant role in desertification control. In this study, we assembled the complete chloroplast genome of *E. rhytidosperma* for the first time, revealing a typical quadripartite structure. The chloroplast genome was 109,545 base pair (bp) long with a GC content of 36.8%, encoding 118 complete genes. Phylogenetic reconstruction resolved *E. rhytidosperma*, *E. minuta*, *E. likiangensis*, *E. equisetina*, *Ephedra gerardiana*, *E. fedtschenkoae*, and *E. monosperma* formed a monophyletic group, indicating their close relationship. The chloroplast genome of *E. rhytidosperma* establishes a good foundation for further genetic and genomic studies of the *Ephedra*.

## 1. Introduction

*Ephedra rhytidosperma* Pachomova (Pachomova 1967), a typically dioecious small shrub belonging to the genus *Ephedra* (Ephedraceae), is distributed in Ningxia, where it thrives on hillsides and proluvial fan (Fu et al. 1999; Hollander et al. 2010; Shi et al. 2022). As an ecologically significant species, it plays a vital role in desertification control through its remarkable sand-binding capacity, attributed to its well-developed root system with powerful resistance to drought, cold, and sand burial (Han et al. 2022) It has also long been an important medicinal plant in China. Containing a plethora of chemical components, it can be used to treat a variety of diseases including cold, asthma, hay fever, and urticaria (Barker and Antia 2007; Mei et al. 2021). Despite these important attributes, the species remains understudied compared to other Ephedraceae members. In particular, the chloroplast genome of *E. rhytidosperma* remains unreported.

Chloroplasts are semi-autonomous organelles essential to plant cells (Sato et al. 1999; Hollingsworth et al. 2016). They possess their own genetic material, namely the chloroplast genome (Li et al. 2015; Daniell et al. 2016; Zhang et al. 2018; Ran et al. 2024). In most plants, chloroplast genome exhibits maternal inheritance and is composed of circular double-stranded DNA molecules ranging from 70 to 210 kb, contains approximately 130 genes (Hu et al. 2016; Guo et al. 2017; Yang et al. 2022). Due to its stable structure and conservative gene content, the chloroplast genome plays a vital role in studying plant origin (Yisilam et al. 2025), phylogenetic relationships (Kan et al. 2024; Yisilam et al. 2025), species identification (Quan et al. 2024), and classification (Gu et al. 2023), serving as a key tool for investigating evolutionary relationships among plant species (Zhang et al. 2012, 2018; Xie et al. 2022).

Here, we report the complete chloroplast genome of *E. rhytidosperma*, the first such assembly for this species. Our work characterizes its genomic structure and resolves the phylogenetic placement of *E. rhytidosperma*, offering valuable data to support taxonomy and evolutionary studies in *Ephedra*.

## 2. Materials

**Figure 1.**
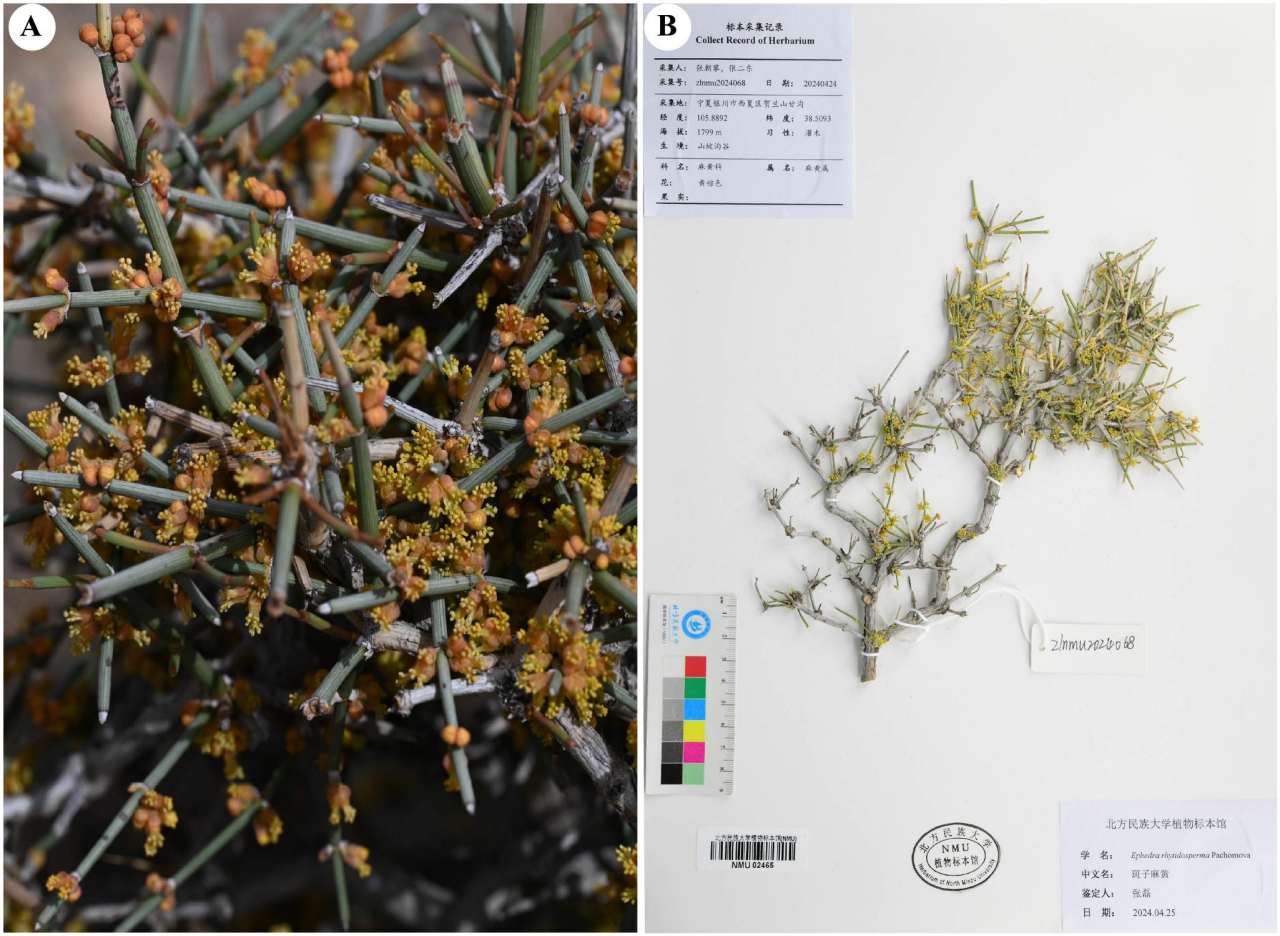
The photos of *E. rhytidosperma*. (A) Whole plant and flowers (photographed by Dr. Lei Zhang). (B) Herbarium of *E. rhytidosperma* (plant is short and nearly pad-like, leaves opposite, seeds 2, exceeding bracts, prominently longitudinally ridged).

Healthy fresh stems of *E. rhytidosperma* were collected from Helan Mountain (Yinchuan, Ningxia, China; coordinates: 105.9058E, 38.9738N) (by Lei Zhang: zhangsanshi-0319@163.com) (Figure 1), and desiccated using silica gel. In addition, collected the branches with flowers or fruits to prepare voucher specimens. The voucher specimen was archived in the Herbarium of North Minzu University (NMU: https://www.cvh.ac.cn/ins/info.php?code=NMU) with an accession number of NMU02465.

## 3. Methods

Total genomic DNA was isolated with a modified CTAB method (Doyle and Doyle 1987). Sequencing libraries were prepared with the NEBNext DNA Library Kit, during which genomic DNA was fragmented into 350 base pair (bp). High-throughput sequencing was subsequently carried out on the Illumina NovaSeq 6000 platform (San Diego, CA) with 150 bp paired-end read length. We acquired 6.2 gigabases (Gb) of high-quality data after adapter trimming.

For chloroplast genome assembly, the *de novo* assembly was implemented in NOVOPlasty 4.3.3 (Dierckxsens et al. 2017) with the specified parameters: k-mer = 39 and genome range 120,000–200,000 bp, using the CPG of *Ephedra intermedia* (MG594471) as a reference seed. Genome annotation was executed through Plann v1.1 (Huang and Cronk 2015) with subsequent manual verification in Geneious v11.0.3 (Kearse et al. 2012). Sequencing depth analysis was quantified using Samtools (Li et al. 2009). The final assembly and annotation of *E. rhytidosperma* was submitted to GenBank (Benson et al. 2013). CPGView website (http://www.1kmpg.cn/cpgview/) was applied to visualize the gene map and the schematic maps of the cis- and trans-splicing genes (Liu et al. 2023).

To establish the phylogenetic placement of *E. rhytidosperma* in *Ephedra*, the chloroplast genomes of 42 representative species were retrieved from NCBI GenBank to reconstruct the chloroplast genome phylogenetic tree, with *Gnetum luofuense* and *G. parvifolium* serving as an outgroup. Protein-coding genes (PCGs) were extracted from the GenBank formatted file containing 43 plastomes using customized Perl scripts, removing start and end codons. A total of 60 PCGs were retained for all species. Each PCG was aligned using PRANK v.130410 (Löytynoja and Goldman 2008) according to the translated amino acid sequences. Phylogenetic reconstruction employed both maximum likelihood (ML) and Bayesian inference (BI) approaches: ML analysis was conducted in RAxML v8.1.24 (Stamatakis 2014) under the GTR + Γ model, while BI analysis in MrBayes v3.2.6 (Ronquist et al. 2012) utilized the GTR + I + G model selected through jModeltest. Final phylogenetic trees were visualized using FigTree v1.4.2 (Rambaut 2012). The results of the comparative analysis of the CPGs were visualized with the mVISTA program (Frazer et al. 2004) and the annotated CPG of *E. intermedia* was used as the reference in the LAGAN mode (Brudno et al. 2003).

## 4. Results

**Figure 2.**
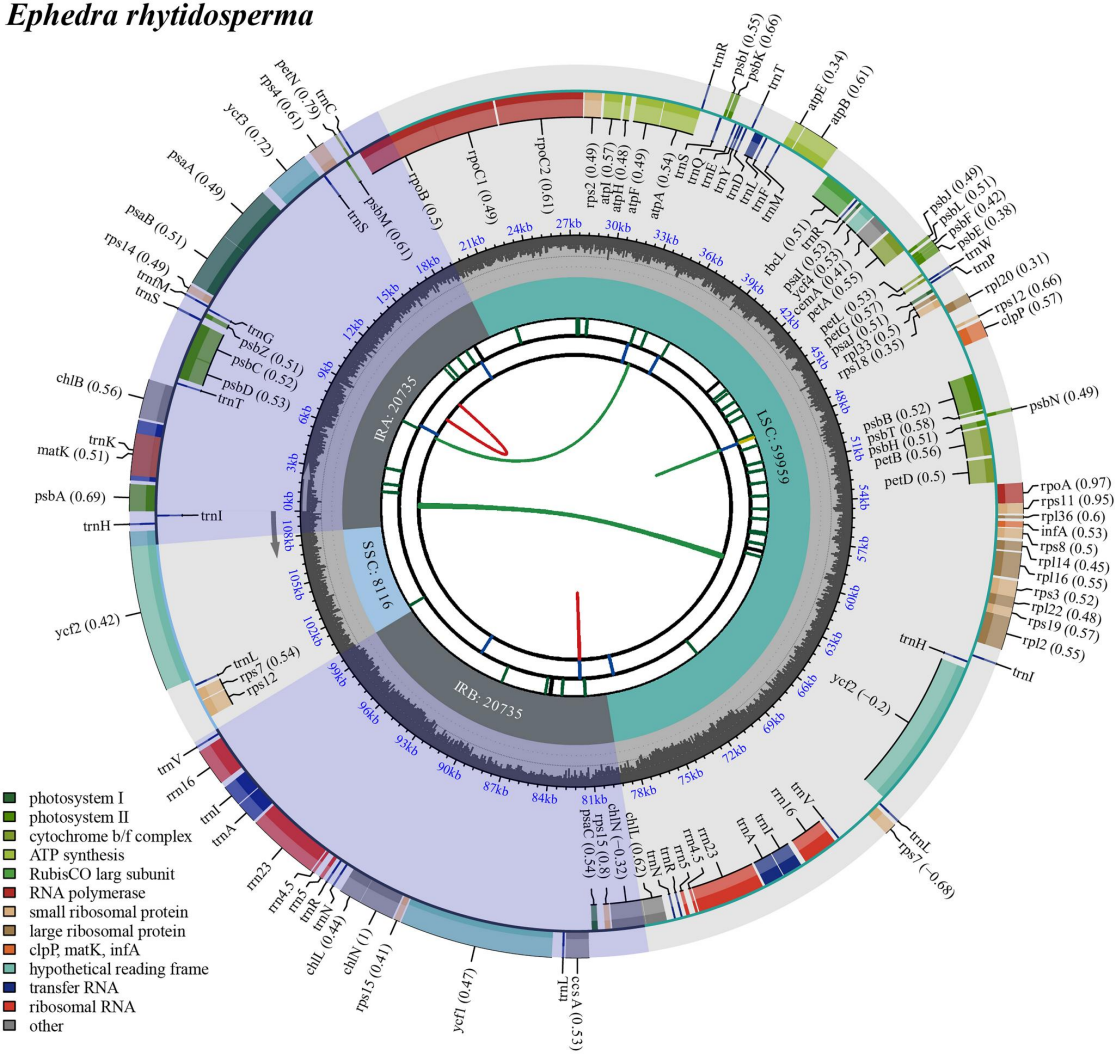
The detailed genome map of *E. rhytidosperma* cp genome. The species name is shown in the top left corner. The map contains six tracks in default. From the center outward, the first track shows the dispersed repeats. The dispersed repeats consist of direct and palindromic repeats, connected with red and green arcs. The second track shows the long tandem repeats as short blue bars. The third track shows the short tandem repeats or microsatellite sequences. The fourth track displays the genome length. The fifth track shows the GC content along the genome, while the sixth track sounds the genes. The gene names are followed by optional information about codon usage bias and color-coded based on their functional classification. The inner genes are transcribed clockwise, and the outer genes are transcribed anticlockwise. The functional type of the genes is shown in the bottom left corner.

Following quality filtering and preprocessing, we obtained at least 4.2 Gb of whole-genome sequencing data were retained. These clean reads were used to assemble high-quality chloroplast genomes through a reference-guided approach. The total chloroplast genome of *E. rhytidosperma* (PV942542) was 109,545 bp long, with sequencing depth analysis revealed maximal, minimal, and average coverage values of 9321×, 27×, and 4478.58×, respectively (Fig. S1). The chloroplast genome exhibited a typical quadripartite structural organization, comprising a large single-copy (LSC) region, two inverted repeat (IR) regions, and a small single-copy (SSC) region (Figure 2 and Figure S2).

**Figure 3.**
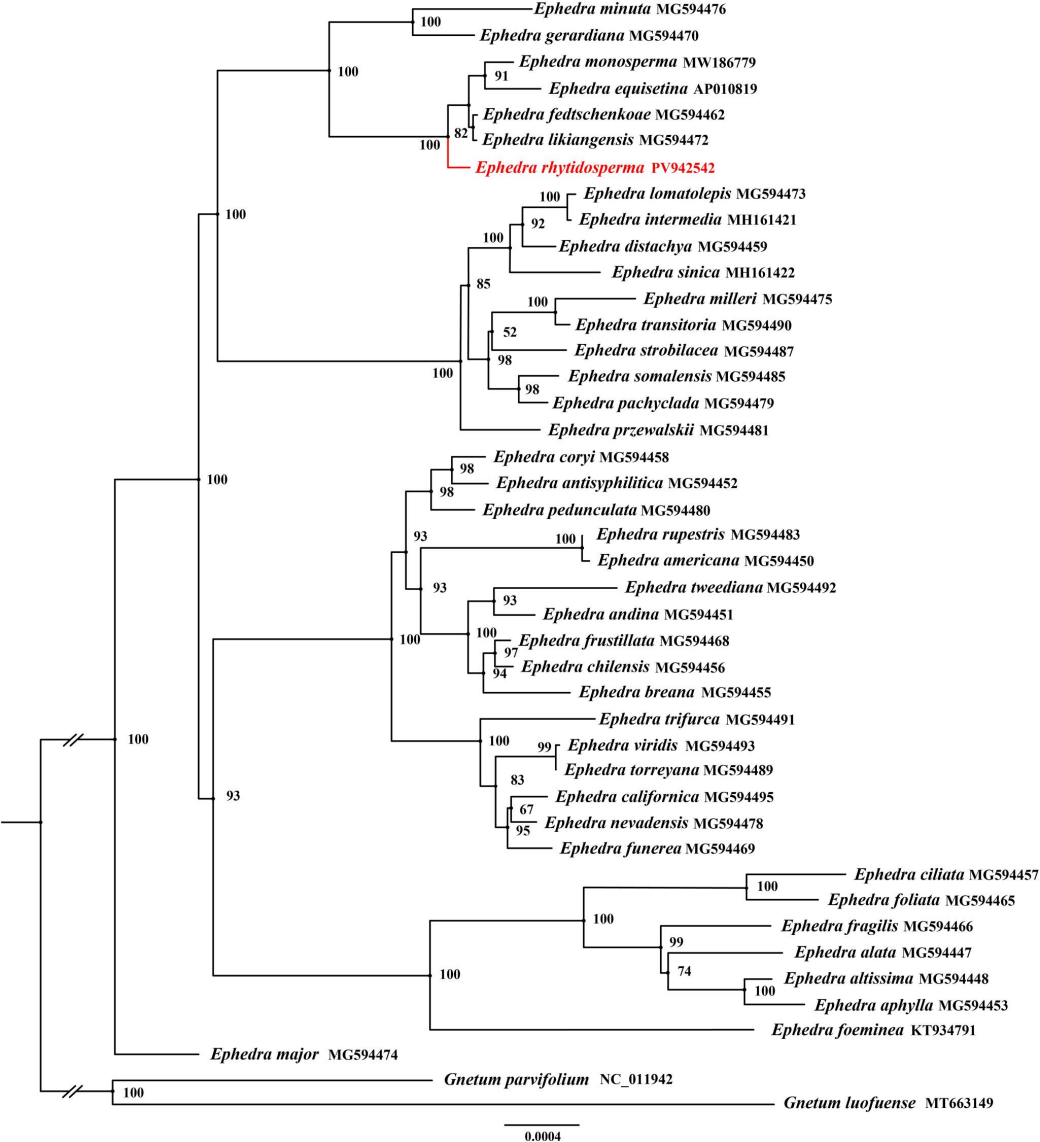
Phylogenetic tree obtained using the maximum likelihood (ML) and Bayesian inference (BI) methods of *Ephedra* species based on based on 60 PCGs. GenBank accession numbers: *Ephedra minuta* MG594476 (Yang et al. 2024), *Ephedra gerardiana* MG594470 (Han et al. 2022), *Ephedra monosperma* MW186779 (Zhao et al. 2021), *Ephedra equisetina* AP010819 (Wu et al. 2009), *Ephedra likiangensis* MG594472 (Rydin et al. 2021), *Ephedra fedtschenkoae* MG594462 (Rydin et al. 2021), *Ephedra lomatolepis* MG594473 (Yang et al. 2024), *Ephedra intermedia* MH161421 (Chen et al. 2019), *Ephedra distachya* MG594459 (Rydin et al. 2021), *Ephedra sinica* MH161422 (Li et al. 2019), *Ephedra milleri* MG594475 (Rydin et al. 2021), *Ephedra transitoria* MG594490 (Rydin et al. 2021), *Ephedra strobilacea* MG594487 (Rydin et al. 2021), *Ephedra somalensis* MG594485 (Zhao et al. 2021), *Ephedra pachyclada* MG594479 (Yang et al. 2024), *Ephedra przewalskii* MG594481 (Rydin et al. 2021), *Ephedra coryi* MG594458 (Rydin et al. 2021), *Ephedra antisyphilitica* MG594452 (Rydin et al. 2021), *Ephedra pedunculata* MG594480 (Rydin et al. 2021), *Ephedra rupestris* MG594483 (Rydin et al. 2021), *Ephedra americana* MG594450 (Rydin et al. 2021), *Ephedra tweediana* MG594492 (Rydin et al. 2021), *Ephedra andina* MG594451 (Rydin et al. 2021), *Ephedra frustillata* MG594468 (Rydin et al. 2021), *Ephedra chilensis* MG594456 (Rydin et al. 2021), *Ephedra breana* MG594455 (Rydin et al. 2021), *Ephedra trifurca* MG594491 (Rydin et al. 2021), *Ephedra viridis* MG594493 (Rydin et al. 2021), *Ephedra torreyana* MG594489 (Rydin et al. 2021), *Ephedra californica* MG594495 (Rydin et al. 2021), *Ephedra nevadensis* MG594478 (Rydin et al. 2021), *Ephedra funerea* MG594469 (Rydin et al. 2021), *Ephedra ciliata* MG594457 (Rydin et al. 2021), *Ephedra foliata* MG594465 (Rydin et al. 2021), *Ephedra fragilis* MG594466 (Rydin et al. 2021), *Ephedra alata* MG594447 (Rydin et al. 2021), *Ephedra altissima* MG594448 (Rydin et al. 2021), *Ephedra aphylla* MG594453 (Rydin et al. 2021), *Ephedra foeminea* KT934791 (Li et al. 2019), *Ephedra major* MG594474 (Rydin et al. 2021), *Gnetum parvifolium* NC_011942 (Chang et al. 2020), *Gnetum luofuense* MT663149 (Yang et al. 2024), and *Ephedra rhytidosperma* PV942542.

The chloroplast genome of *E. rhytidosperma* annotation identified 118 complete genes (Table S1), comprising 73 PCGs, eight ribosomal RNA genes (rRNAs), and 37 tRNA genes (tRNAs). Additionally, the genome contained one trans-splicing gene (Fig. S3) and seven cis-splicing genes (Fig. S4), with an overall GC content of 36.8%. Phylogenetic reconstruction using both ML and BI methods robustly placed *E. rhytidosperma* within the *Ephedra* (Figure 3 and Figure S5). In these trees, the 41 *Ephedra* species dividing into five strongly supported clades (BS = 100%; PP = 1) (Figure 3 and Figure S5). Notably, *E. rhytidosperma* clustered with *E. minuta*, *Ephedra gerardiana*, *E. monosperma*, *E. equisetina*, *E. fedtschenkoae*, and *E. likiangensis* (BS = 100%; PP = 1), indicating a close evolutionary relationship between these species.

## 5. Discussion and conclusions

In this study, we assembled the CPG of *E. rhytidosperma* for the first time, revealing a typical quadripartite structure consisting of a pair of IRs, a LSC region, and a SSC region. The assembled genome has a total length of 109,545 bp, contains 118 complete genes, and exhibits a GC content of 36.8%. These characteristics are consistent with those reported for other *Ephedra* species (Chen et al. 2019; Han et al. 2022).

The cp genomes have become a central focus in molecular biology research due to their significant potential for resolving phylogenetic relationships among higher plants. In this study, phylogenetic analyses using BI and ML methods revealed that *Ephedra* cp genomes form five strongly supported main clades (BS = 100%; PP = 1), a result consistent with previous studies (Chen et al. 2019; Zhao et al. 2021). Notably, *E. rhytidosperma*, *E. minuta*, *E. gerardiana*, *E. monosperma*, *E. equisetina*, *E. fedtschenkoae*, and *E. likiangensis* form a distinct monophyletic group. The sequence alignment of the CPGs of the above seven *Ephedra* species indicated high sequence similarity among the CPGs of the seven *Ephedra* species (Fig. S6). However, sequence divergence in non-coding regions was greater than that in coding regions. The faster evolutionary rate in non-coding regions provides abundant raw material for natural selection. To further advance our understanding of *E. rhytidosperma*, future studies should prioritize a comprehensive analysis of its codon usage bias and population-level genomic diversity. Such investigations are key to elucidating structural variations in its chloroplast genome. Moreover, expanding the repertoire of complete chloroplast genomes across the *Ephedra* genus will be crucial for clarifying the evolutionary dynamics of this ecologically significant group.

## Supplementary Material

Supplementary File.docx

## Data Availability

The *Ephedra rhytidosperma* chloroplast genome sequence data that support the findings of this study are openly available in NCBI (https://www.ncbi.nlm.nih.gov/) under the accession no. PV942542. The associated BioProject, SRA, and Bio-Sample numbers are PRJNA1292112, SRR34590523, and SAMN49990842, respectively.
