# Supplementary material for: The complete chloroplast genome sequence of the endangered species: Ephedra rhytidosperma (Ephedraceae): insights into genome features and evolutionary relationships
Source: Mitochondrial DNA B Resour. 2025 Nov 5;10(12):1093–8. doi: 10.1080/23802359.2025.2582546 (PMC12599160; doi:10.1080/23802359.2025.2582546)
Supplement: Supplementary File.docx [file TMDN_A_2582546_SM7729.docx]

The complete chloroplast genome sequence of the endangered species: *Ephedra rhytidosperma* (Ephedraceae): insights into genome features and evolutionary relationships

Xiulian Liao^1^, Jinfeng Niu^2^, Yan Wang^1^, Yuqing Wei^1^, Lei Zhang^1*^

^1^ Key Laboratory of Ecological Protection of Agro-Pastoral Ecotones in the Yellow River Basin, National Ethnic Affairs Commission of the People’s Republic of China, School of Biological Science & Engineering, North Minzu University, Yinchuan 750021, China

^2^ Ningxia State-owned Forest Farms and Forest Tree Seedling Work General Stations, Yinchuan 750021, China

***Corresponding author**: Lei Zhang, zhangsanshi-0319@163.com

**ORCID**: Lei Zhang; https://orcid.org/0000-0001-5301-4658

Table S1 Genes present in the *E. rhytidosperma* cp genome

| Classifcation | Gene family | Gene name |
| --- | --- | --- |
| Photosynthesis related genes | Photosystem l (5) | *psa*A, *psa*B, *psa*C, *psa*I, *psa*J |
|  | Photosystem ll (16) | *psb*A, *psb*B, *psb*C, *psb*D, *psb*E, *psb*F, *psb*H, *psb*I, *psb*J, *psb*K, *psb*L, *psb*M, *psb*N, *psb*T, *psb*Z |
|  | Cytochrome b/f complex (6) | *pet*A, *pet*B*, *pet*D*, *pet*G, *pet*L, *pet*N |
|  | *atp* synthase (6) | *atp*A, *atp*B, *atp*E, *atp*F*, *atp*H, *atp*I |
|  | NADH-dehydrogenase (11) | *ndh*A, *ndh*B, *ndh*C, *ndh*D, *ndh*E, *ndh*F, *ndh*G, *ndh*H, *ndh*I, *ndh*J, *ndh*K |
|  | Subunit of rubisco (1) | *rbc*L |
| Transcription and translationrelated genes | *rrn*A gene (8) | *rrn*16(x2), *rrn*23(x2), *rrn*4.5(x2), *rrn*5(x2) |
|  | *trn*A gene (37) | *trn*K-UUU*, *trn*T-GGU, *trn*S-UGA, *trn*G-UCC, *trn*fM-CAU(x2), *trn*S-GGA, *trn*C-GCA, *trn*R-UCU, *trn*S-GCU, *trn*Q-UUG, *trn*E-UUC, *trn*Y-GUA, *trn*D-GUC, *trn*T-UGU, *trn*F-GAA, *trn*M-CAU, *trn*R-CCG, *trn*W-CCA, *trn*P-UGG, *trn*I-CAU, *trn*H-GUG(x2), *trn*L-CAA(x2), *trn*V-GAC(x2), *trn*R-ACG(x2), *trn*N-GUU(x2), *trn*L-UAG, *trn*A-UGC*, *trn*L*, *trn*I(x2)*, *trn*A* |
|  | Small subunit of ribosome (15) | *rps*11, *rps*12**(x2), *rps*14, *rps*15(x2), *rps*18, *rps*19, *rps*2, *rps*3, *rps*4, *rps*7(x2), *rps*8, *rps*16 |
|  | Large subunit of ribosome (9) | *rpl*14, *rpl*16*, *rpl*2*, *rpl*20, *rpl*22, *rpl*33, *rpl*36, *rpl*23, *rpl*32 |
|  | DNA dependent RNA polymerase (4) | *rpo*A, *rpo*B, *rpo*C1*, *rpo*C2 |
| Others genes | *mat*urase (1) | *mat*K |
|  | Acetyl-CoA-carboxylase (1) | *acc*D |
|  | c-type cytochrom synthesis gene(1) | *ccs*A |
|  | Chloroplast envelop membrane protein (1) | *cem*A |
|  | Protease (1) | *clp*P |
| Unknown function genes  Hypothetical | Hypothetical chloroplast conservedopen reading frames (6) | *ycf*1, *ycf*2(x2), *ycf*3**, *ycf*4 , *ycf*15 |

# represents a gene with one intron, represents a gene with two introns,(x2) repeat genes


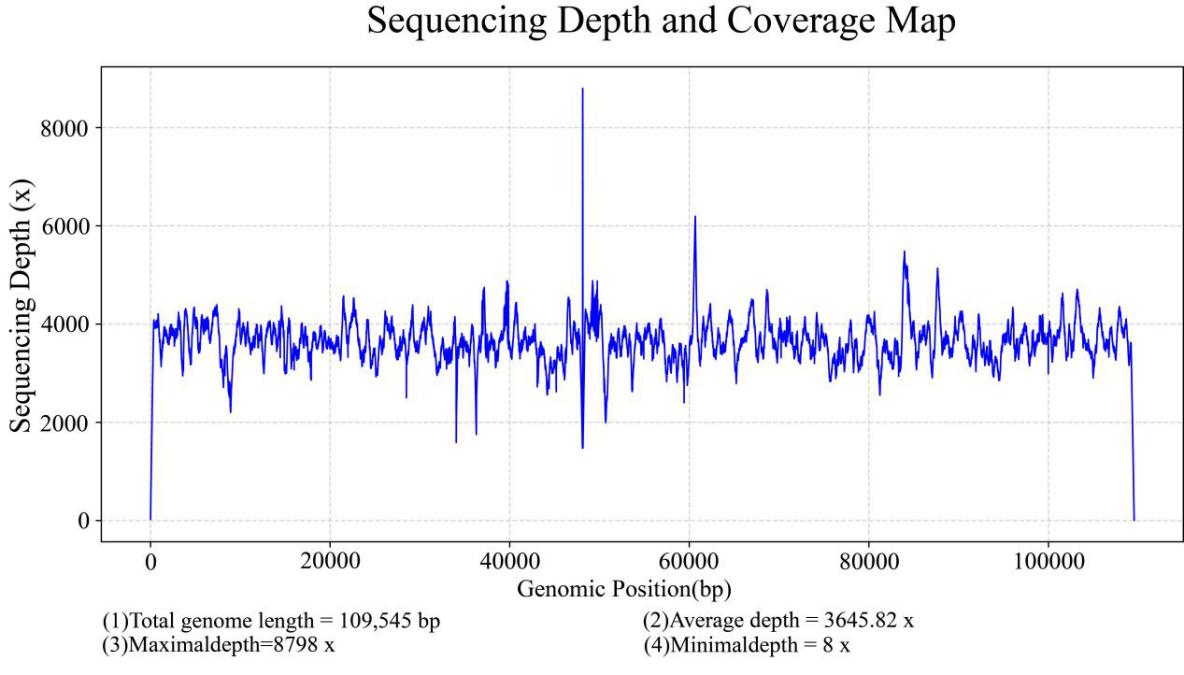


**Figure S1** Coverage depth distribution of the *E. rhytidosperma*.


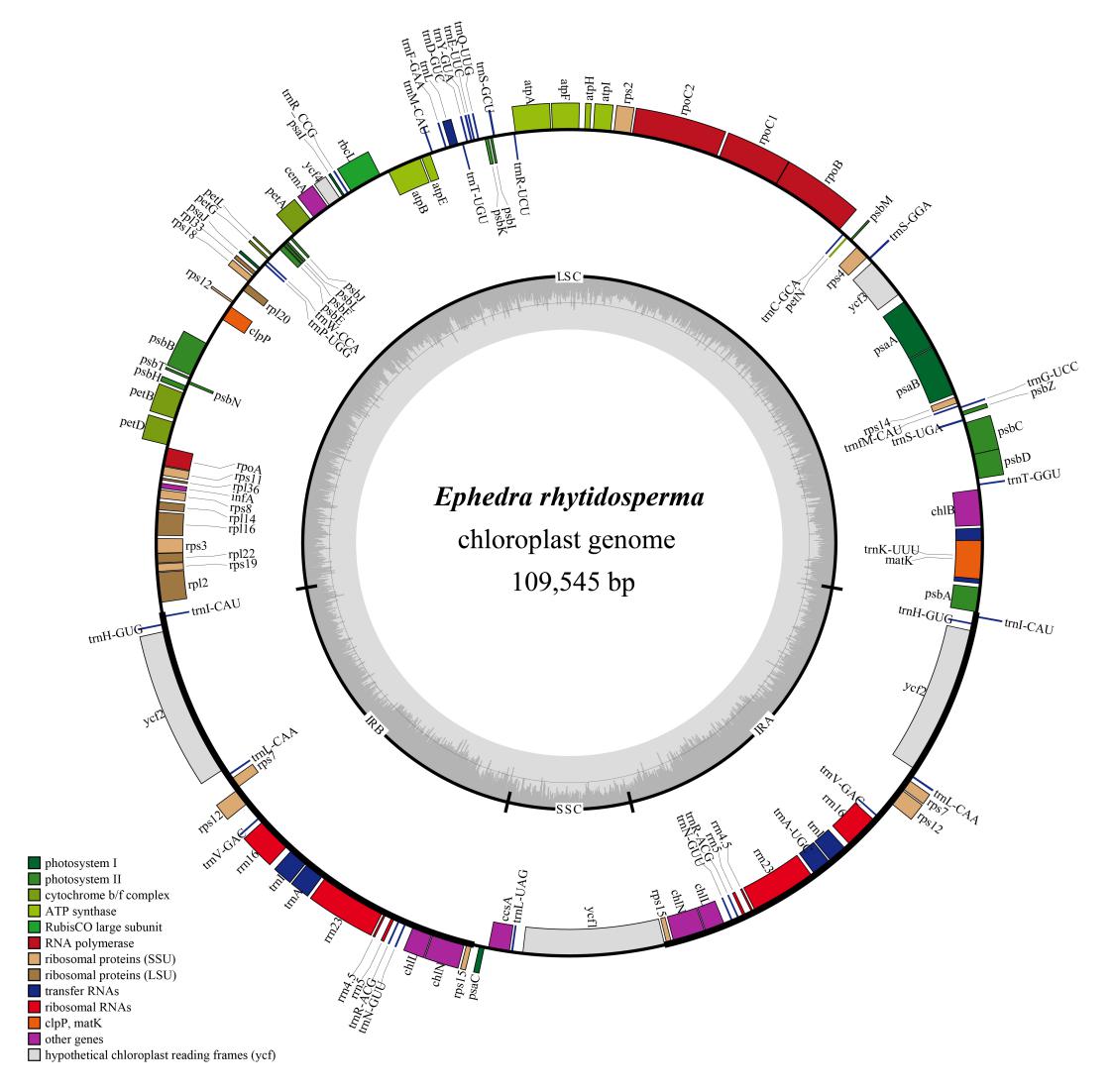


**Figure S2** The genome map of *C. patelliforme* cp genome. GC content (light gray) is shown in the inside track. Gene models including protein-coding genes, tRNA genes and rRNA genes are shown with various colored boxes in the outer track.


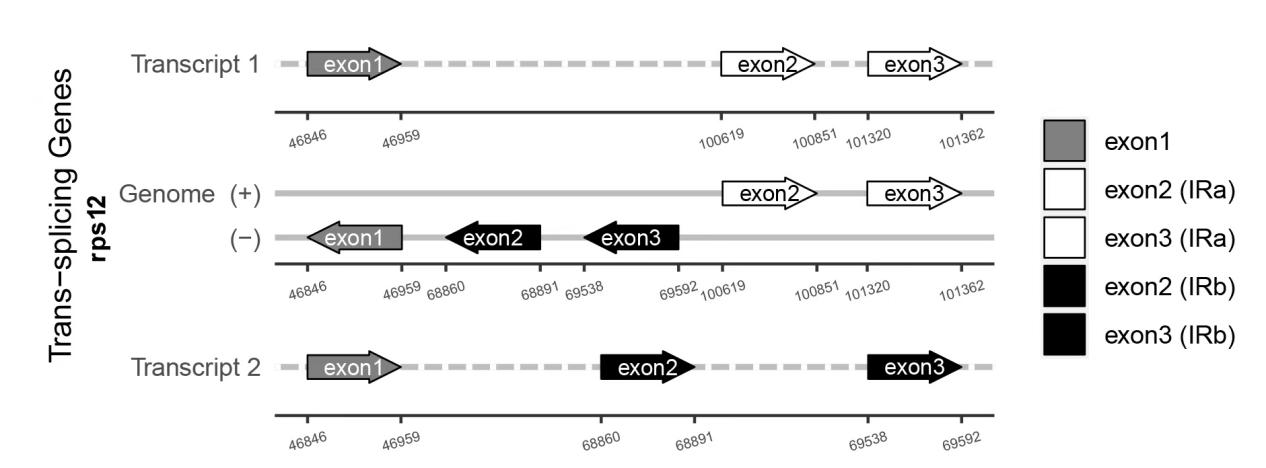


**Figure S3** Structure of trans-splicing genes in the *E. rhytidosperma*.


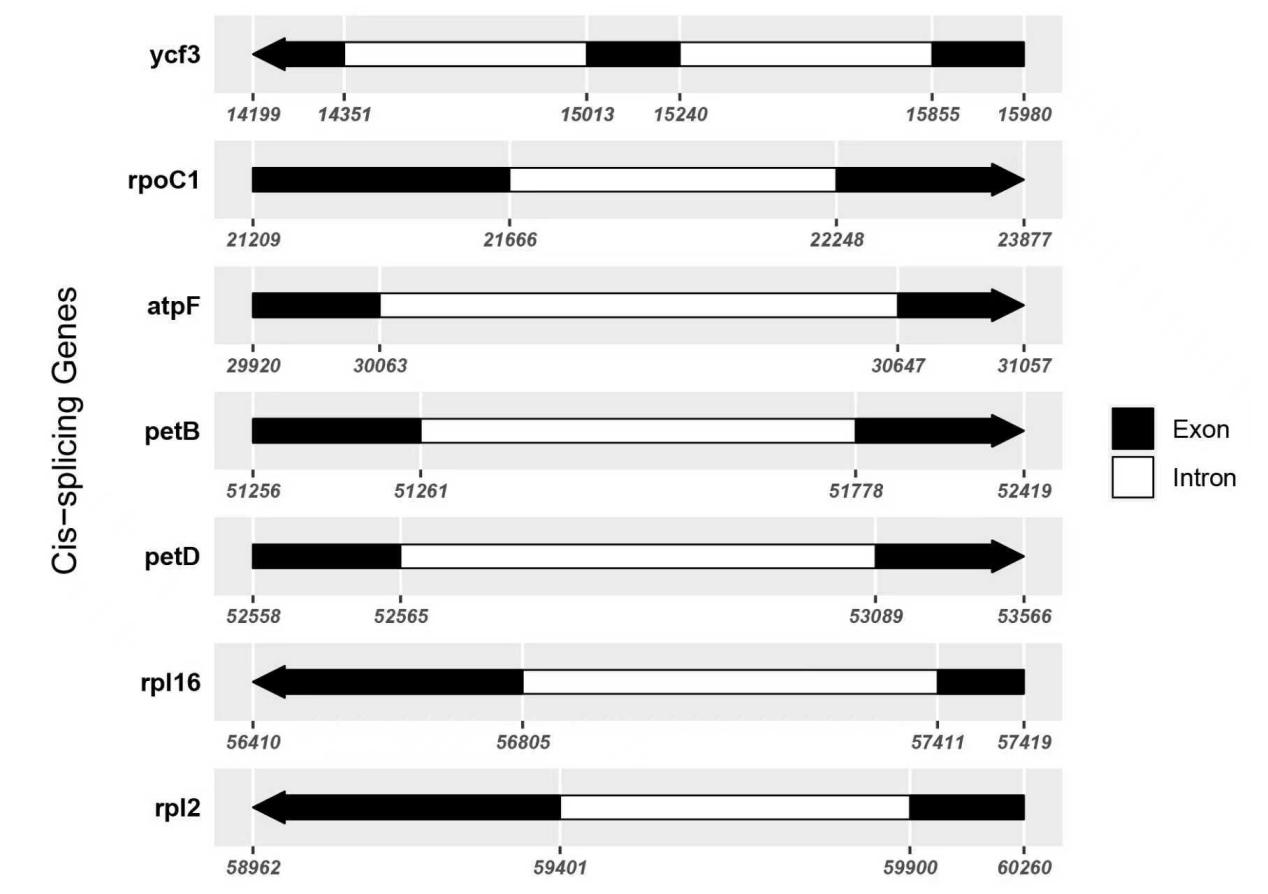


**Figure S4** Structure of Cis-splicing genes in the *E. rhytidosperma*.


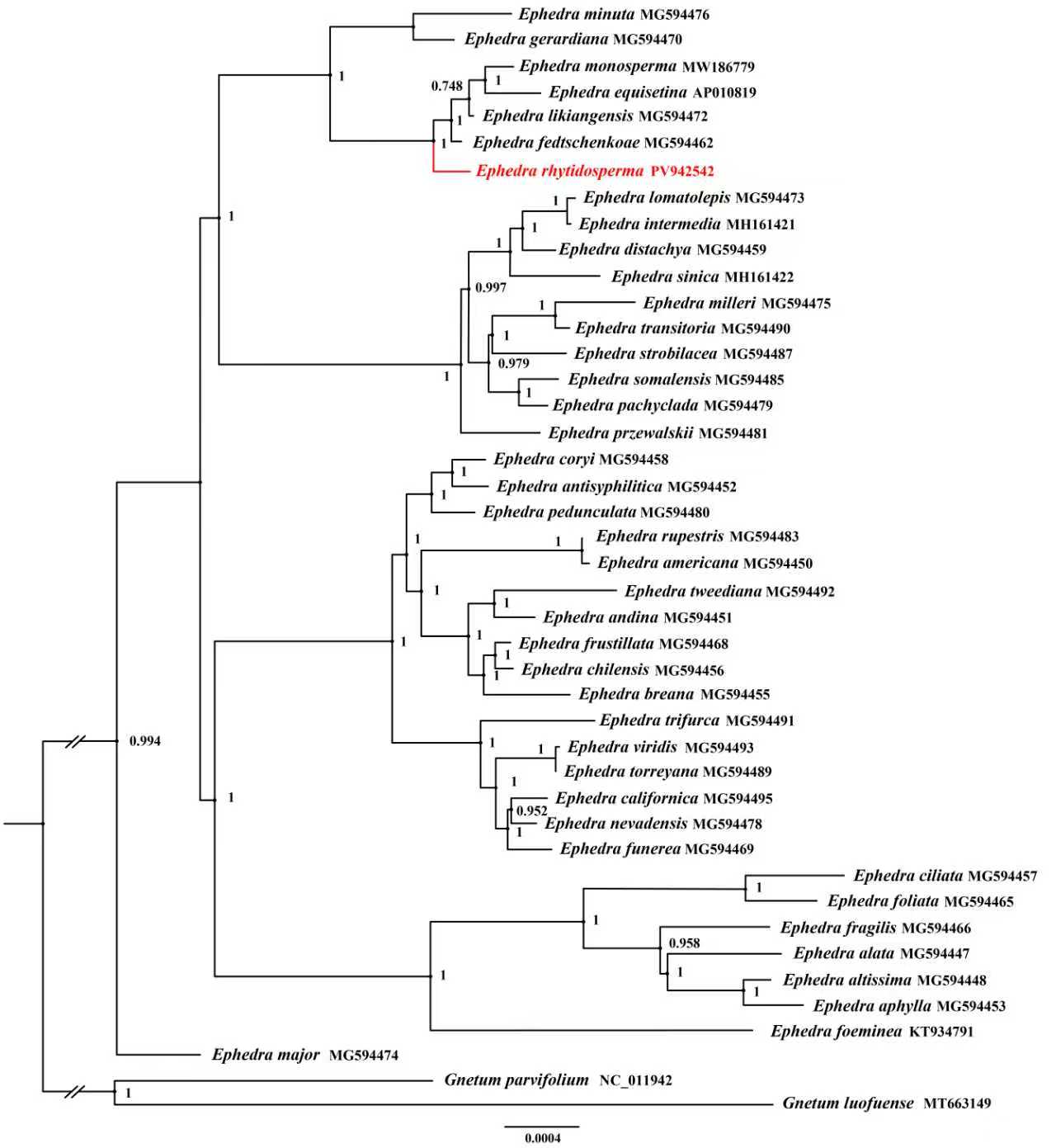


Figure S5 Phylogenetic tree obtained using the bayesian inference (BI) methods of *Ephedra* species based on 60 PCGs.


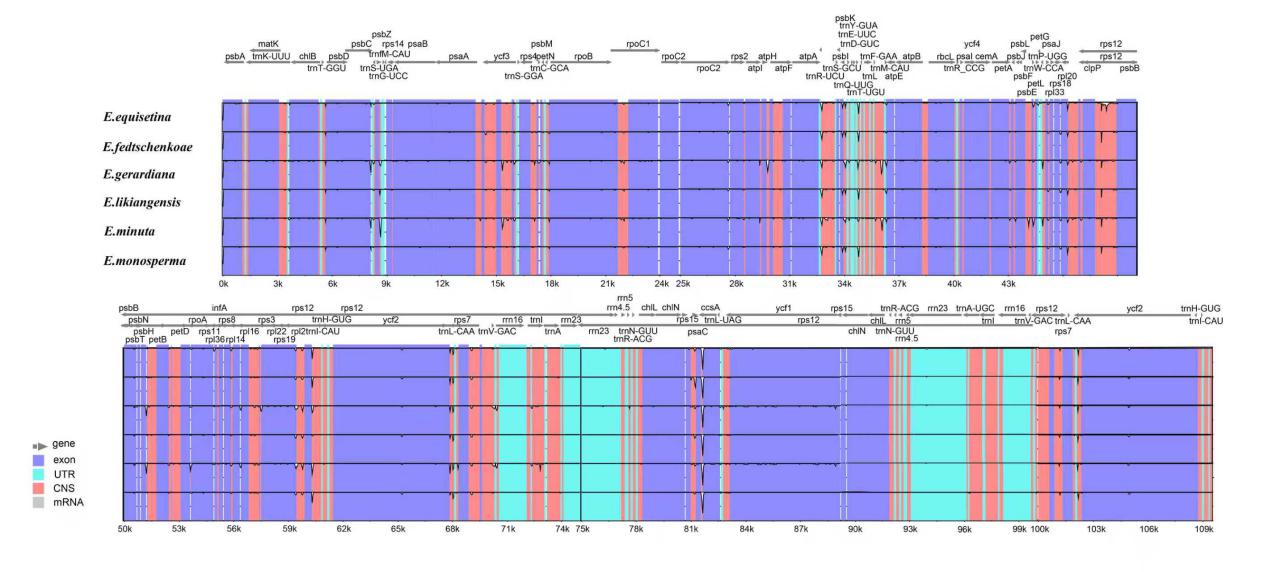


Figure S6 Sequence alignment of the CPGs of seven *Ephedra* species.
